# Editorial: Plant-Pest Interactions Volume II: Hemiptera

**DOI:** 10.3389/fpls.2021.748999

**Published:** 2021-09-13

**Authors:** Felix Ortego, Colette Broekgaarden, Takeshi Suzuki, George Broufas, Guy Smagghe, Isabel Diaz

**Affiliations:** ^1^Centro de Investigaciones Biologicas Margarita Salas, CSIC, Madrid, Spain; ^2^KeyGene N.V., Wageningen, Netherlands; ^3^Graduate School of Bio-Applications and Systems Engineering, Tokyo University of Agriculture and Technology, Koganei, Japan; ^4^Department of Agricultural Development, Faculty of Agricultural Sciences and Forestry, Democritus University of Thrace, Komotini, Greece; ^5^Department of Plants and Crops, Ghent University, Ghent, Belgium; ^6^Centro de Biotecnología y Genómica de Plantas, Universidad Politécnica de Madrid (UPM)-Instituto Nacional de Investigación y Tecnología Agraria y Alimentaria, Madrid, Spain; ^7^Departamento de Biotecnología-Biología Vegetal, Escuela Técnica Superior de Ingeniería Agronómica, Alimentaria y de Biosistemas, Madrid, Spain

**Keywords:** hemipteran, plant defence, resistance, multiple interactions, insects

Plant-pest interactions involve multi-faced processes encompassing a complex network of molecules, signals, and pathways to overcome defences developed by each other. Insects end is to obtain nutrients from their hosts and to assure a safe place for oviposition. Plants respond to insect infestation by triggering defence mechanisms including the development of physical barriers to hamper pest access and compounds with antinutritional, deterrent, repellent, and toxic properties to interfere with the physiology and behaviour of the herbivore. In turn, insects reply by developing strategies to avoid plant defences. In a second round, plants counter-attack implementing emergency responses. Progress, particularly on the molecular analyses of these relationships, has been widely published in recent years (reviewed by Santamaria et al., 2018; Stahl et al., 2018; Erb and Reymond, 2019; Wilkinson et al., 2019; Hamann et al., 2021).

This Research Topic is addressed in a special issue on plant-pest interactions which has been divided into three volumes based on the pest order. This volume II is focussed on hemipteran species, an extensive group of insect piercing-sucking species (e.g., aphids, whiteflies, stinkbugs, leafhoppers, and planthoppers) with a great impact on agricultural production worldwide. Phytophagous hemipteran may directly hurt plants but the major threat is due to the role of same species as vectors of plant pathogens. The understanding of mechanisms and molecular factors that participate in the plant-hemipteran interplay, mainly focused on aphids, has increased in the last decade (Koch et al., 2016; Shah and Walling, 2017; Nalam et al., 2019). The eight articles included in volume II add novel insights at the ecophysiological and molecular levels on plant-hemipteran interactions.

Deciphering of the plant defence responses in the interaction with aphids have been the objective of several articles. Pincebourde and Ngao have investigated the impact of the green apple aphid (*Aphis pomi*) on the leaf physiology of apple trees, across a range of leaf age. Results revealed that *A. pomi* enhanced assimilation and transpiration rates, stomatal conductance and internal CO_2_ concentration in apple leaves up to about the age of 30 days, and then, moved upward to younger leaves. After aphid migration, the carbon content came back to the level of non-infested leaves but the gas exchange patterns still differed, while the nitrogen/carbon ratio never reached the level of non-infested plants. Thus, the gas exchange may explain how plants could support moderate insect pressure. This relation between the leaf age and aphid infestation was also highlighted by Singh et al.. After evaluating the preference and feeding behaviour of the bird cherry-oat aphid *Rhopalosiphum padi* among several accessions of *Triticum turgidum* and a domesticated durum wheat cultivar. They conclude that that: (i) the position of the leaf (leaf age) defined the aphid performance; and (ii) the trichome density, and particularly the induction of benzoxazinoids in infested leaves were the main factors to determined aphid resistance. Likewise, Gyan et al. reported that those accessions of tef (*Eragrotis tef* ) with the higher number of trichomes presented a reduced *R. padi* progeny. Moreover, the volatile profile of tef infested plants presented similar defence responses as other Poaceae species. To control aphids, previous data had shown that *Rag* genes conferred resistance to soybean against Aphis glycyine and these genes were deployed in commercial cultivars (Hesler et al., 2013). However, soybean plants carrying the *Rag5* gene were aphid resistant in whole plant assays but not in detached leaf assays. Joshi et al., confirmed previous findings and demonstrated that the resistance was located in the stem and correlated with the high kaempferol content in this tissue.

Plant-pest interactions can be influenced by both abiotica and biotic factors. Under climate change scenarios associated with high temperatures, increased atmospheric CO_2_ levels and elevated nitrogen deposition, a greater food consumption by phytophagous arthropods is expected (Bellard et al., 2012; Hamann et al., 2021). In this context, Carreras Navarro et al. have analysed the effect of different N application rates and CO_2_ levels on wheat growth and *R. padi* performance. These authors found that elevated CO_2_ treatments negatively correlated with wheat N content and aphid fecundity, whereas greater N applications increased both the plant N content and the aphid fecundity. So, environmental parameters determine plant and insect development, and consequently, grain yield and quality. Nevertheless, not only abiotic elements modified plant defences against pest, biotic factors also have a big impact. This has been demonstrated by Dove et al. who have analysed the microbiomes of needle, branch, root, and rhizosphere of two hemlock species, *Tsuga canadiensis* and *T. sieboldii*, with low and high population levels of the hemlock woody adelgied *Adelges tsugae*, respectively. Their findings highlighted that the variation between microbiomes was better explained by the host species or the plant tissue/organ habitats than by the invasive insect levels. In the same research line, another article by Mbaluto et al. reported the impact of a root-knot nematode *Meloidogyne incognita* on tomato leaf responses induced by the potato aphid *Macrosiphum euphorbiae*, and conversely the aphid-infested tomato responses to the nematode. Results revealed that nematode and aphid triggered different local and systemic defence responses and an asymmetrical interaction between them when plants were co-infested. Aphid feeding did not systematically alter the nematode-induced defences in roots, and *M. incognita* determined root defences regardless of the aphid.

Finally, a nice review by Naalden et al. updated the current knowledge on whitefly effectors, their plant targets, their function of the defence transduction pathways and their final impact on plant resistance.

The information reported in this volume II on plant-pest interaction, has added key elements in plant-hemipteran insect interplay, but further research is required to get a full understanding and for exploiting natural defence mechanisms in agriculture.

## Author Contributions

ID wrote the Editorial with contributions from all co-authors. All authors have acted as co-editors of this special issue and approved the submitted version.

## Conflict of Interest

The authors declare that the research was conducted in the absence of any commercial or financial relationships that could be construed as a potential conflict of interest.

## Publisher's Note

All claims expressed in this article are solely those of the authors and do not necessarily represent those of their affiliated organizations, or those of the publisher, the editors and the reviewers. Any product that may be evaluated in this article, or claim that may be made by its manufacturer, is not guaranteed or endorsed by the publisher.
